# Supplementary material for: Mass spectrometry imaging of phosphatidylcholine metabolism in lungs administered with therapeutic surfactants and isotopic tracers
Source: J Lipid Res. 2021 Jan 14;62:100023. doi: 10.1016/j.jlr.2021.100023 (PMC7961103; doi:10.1016/j.jlr.2021.100023)
Supplement: Supplemental Figures S1–S10 [file mmc1.pdf]

Supporting Information For:

Mass spectrometry imaging of phosphatidylcholine metabolism in lungs administered with therapeutic surfactants and isotopic tracers

Shane R. Ellis[1,2,3]*, Emily Hall[4], Madhuriben Panchal[4,5], Bryn Flinders[1]†, Jens Madsen[6], Grielof Koster[4,5], Ron. M. A. Heeren[1], Howard W. Clark[6,7], Anthony D. Postle[4,5]*

[1]Maastricht MultiModal Molecular Imaging (M4I) Institute, Division of Imaging Mass Spectrometry, Maastricht University, Universiteitssingel 50, 6229ER, Maastricht, The Netherlands

[2]Molecular Horizons and School of Chemistry and Molecular Bioscience, University of Wollongong, Wollongong, New South Wales, 2522, Australia

[3]Illawarra Health and Medical Research Institute, Wollongong, NSW 2522, Australia

[4]Clinical & Experimental Sciences, Faculty of Medicine, University of Southampton, SO16 6YD, UK

[5]National Institute for Health Research Southampton Biomedical Research Centre, University Hospital Southampton, SO16 6YD, UK

[6]Elizabeth Garrett Anderson Institute for Women's Health, Faculty of Population Health Sciences, University College London, London, WC1E 6AU, UK

[7]National Institute for Health Research University College London Hospital Biomedical Research Centre, London, W1T 7DN, UK

*To whom correspondence should be addressed:

sellis@uow.edu.au.

A.D.Postle@soton.ac.uk

†Current address: Hair Diagnostix, Dutch Screening Group, Gaetano Martinolaan 63A, 6229 GS Maastricht, The Netherlands.

Running title: Imaging of PC metabolism in lung using isotope labelling

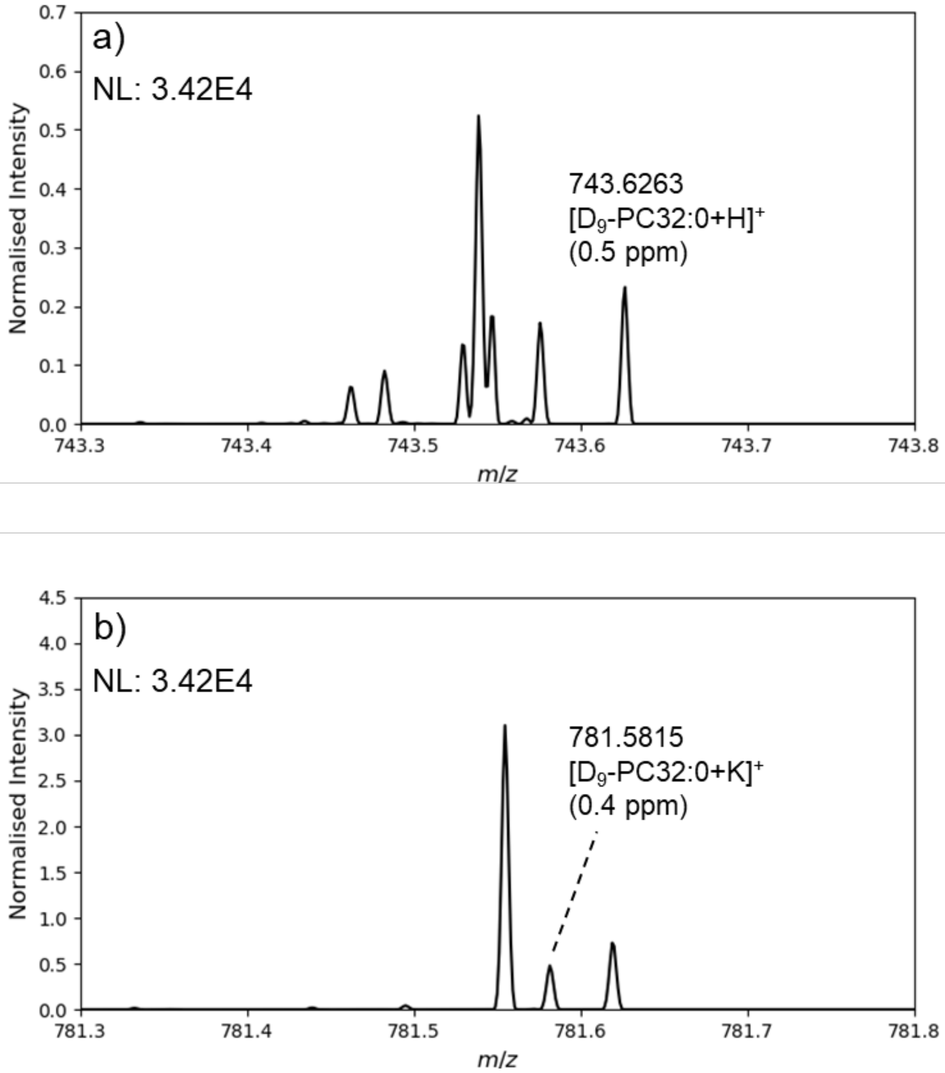

Supplementary Figure S1. Zoomed-in regions of the averaged positive-ion MALDI spectrum acquired from mouse lung tissue dosed with D_9-choline and U-C_{13}-DPPC–containing CHF5633 surfactant (labels administered 12 h prior to sacrifice). Spectra demonstrate the detection of (a) ([D_9-PC32:0+H]$^+$ and (b) ([D_9-PC32:0+K]$^+$). Parts-per-million (ppm) mass errors are indicated in parentheses and spectra are normalised to the base peak intensity (0–100%). The 'NL" value refers to the intensity of the base peak in the full range MS^1 spectrum averaged over the entire measurement region.

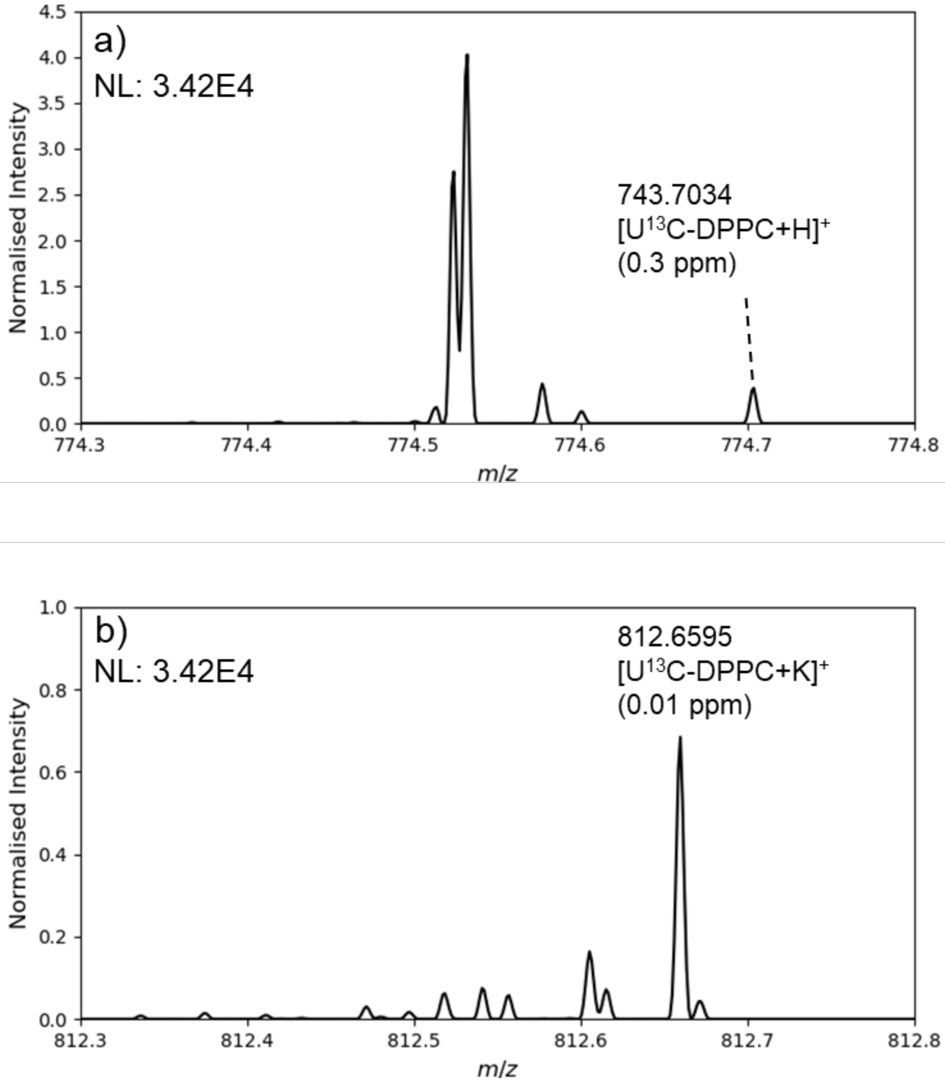

Supplementary Figure S2. Zoomed-in regions of the averaged positive-ion MALDI spectrum acquired from mouse lung tissue dosed with D_9-choline and $U^{13}C$-DPPC–containing CHF5633 surfactant (labels administered 12 h prior to sacrifice). Spectra demonstrate the detection of (a) ($[U^{13}C\text{-DPPC+H}]^+$ and (b) ($[U^{13}C\text{-DPPC+K}]^+$). Parts-per-million (ppm) mass errors are indicated in parentheses and spectra are normalised to the base peak intensity (0–100%). The 'NL" value refers to the intensity of the base peak in the full range MS^1 spectrum averaged over the entire measurement region. DPPC = PC16:0/16:0

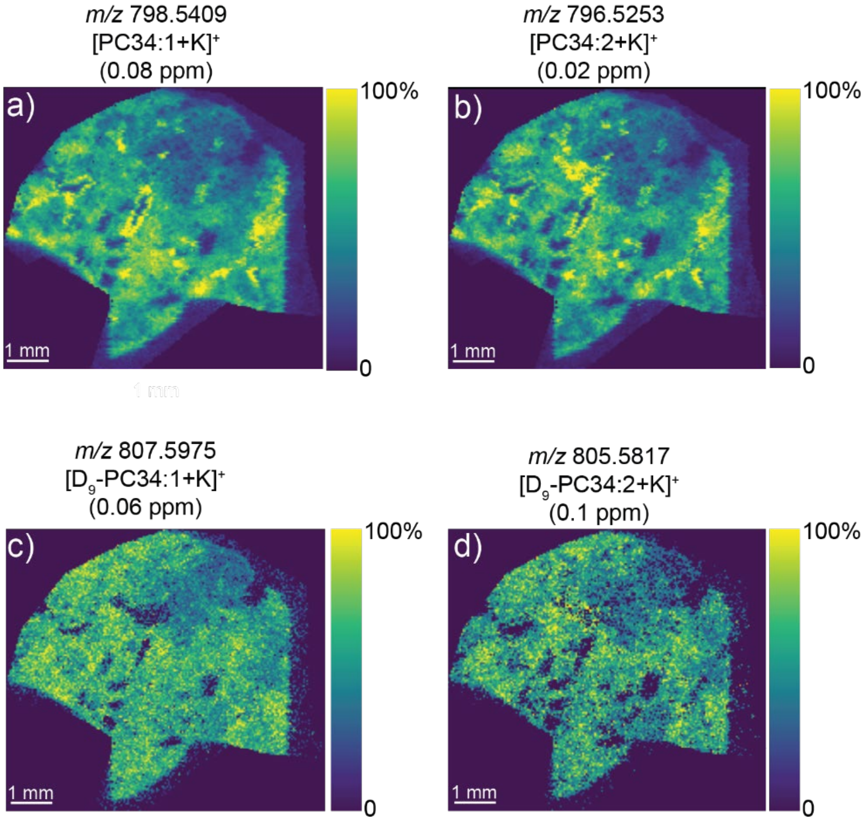

Supplementary Figure S3. Ion distribution images for (a) $[PC34:1+K]^+$ (*m/z* 798.5409), (b) $[PC34:2+K]^+$ (*m/z* 796.5253), (c) $[D_9\text{-}PC34:1+K]^+$ (*m/z* 807.5975) and (d) $[D_9\text{-}PC34:2+K]^+$ (*m/z* 805.5817) obtained from the same tissue as that shown in Figures 2 and 3 in the main text. Parts-per-million (ppm) mass errors are indicated in parentheses. The potassiated adducts of PC34:1 and PC-34:2-related ions were chosen for visualisation to avoid potential isobaric interference with isobaric $[PC36:4+H]^+$ and $[PC36:3+H]^+$. All MSI images were visualised using total ion current normalisation and hotspot removal (high quantile = 99%).

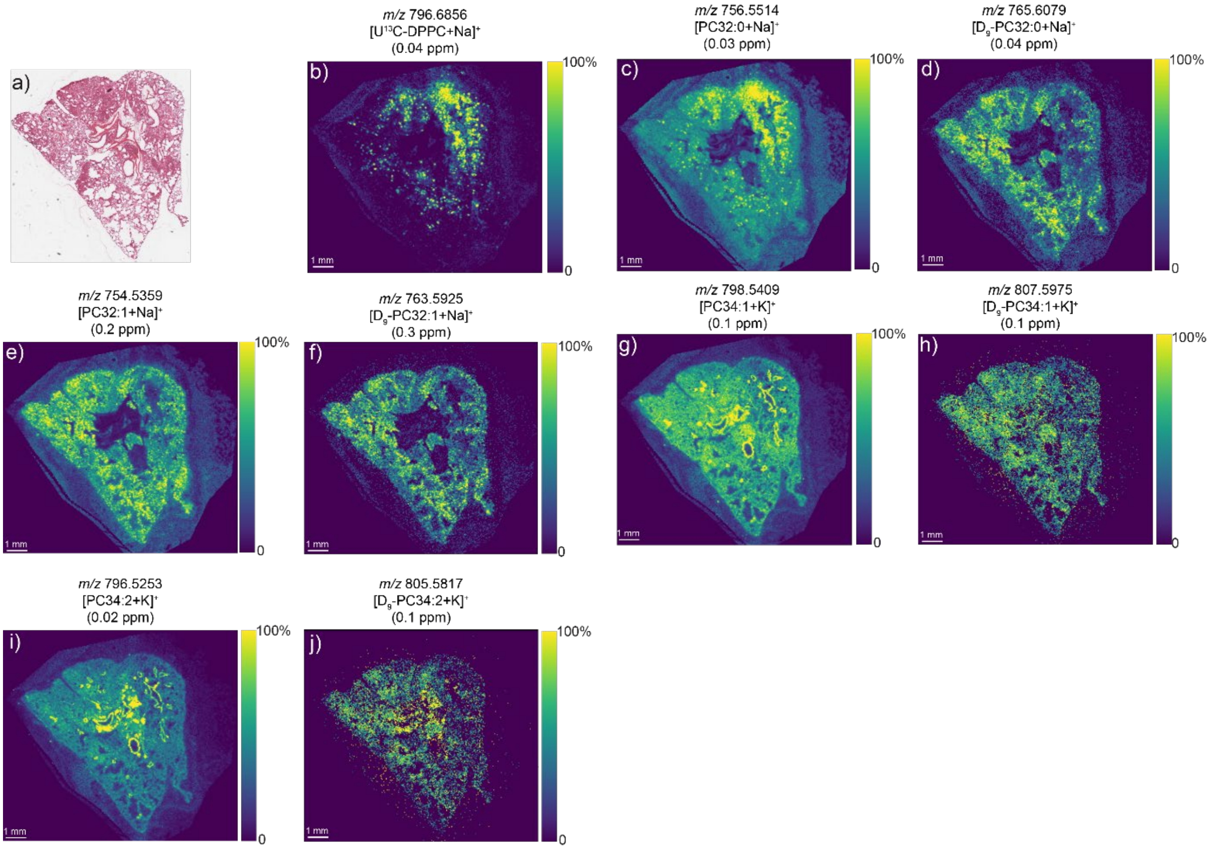

Supplementary Figure S4. MALDI-MSI data from mouse lung tissue administered with D_9-choline and $U^{13}C$-DPPC–containing CHF5633 surfactant (labels administered 6 h prior to sacrifice, same tissue as shown in Figure S4). (a) Optical image of post-MSI H&E-stained tissue section. (b–j) Ion images of (b) *m/z* 796.6856 ($[U^{13}C\text{-DPPC+Na}]^+$), (c) *m/z* 756.5514 ($[PC32{:}0\text{+Na}]^+$), (d) *m/z* 765.6079 ($[D_9\text{-PC32:0+Na}]^+$), (e) *m/z* 754.5359 ($[PC32{:}1\text{+Na}]^+$), (f) *m/z* 763.5925 ($[D_9\text{-PC32:1+Na}]^+$), (g) *m/z* 798.5409 ($[PC34{:}1\text{+K}]^+$), (h) *m/z* 807.5975 ($[D_9\text{-PC34:1+K}]^+$), (i) *m/z* 796.5253 ($[PC34{:}2\text{+K}]^+$) and (j) *m/z* 805.5817 ($[D_9\text{-PC34:1+K}]^+$). The lipid signal observed adjacent to the tissue section is potentially attributable to smearing artefacts during tissue mounting, however it does not alter the on-tissue distributions. Part-per-million (ppm) mass errors are indicated in parentheses. All images were visualised using total-ion-current normalisation and hotspot removal (high quantile = 99%). DPPC = PC16:0/16:0.

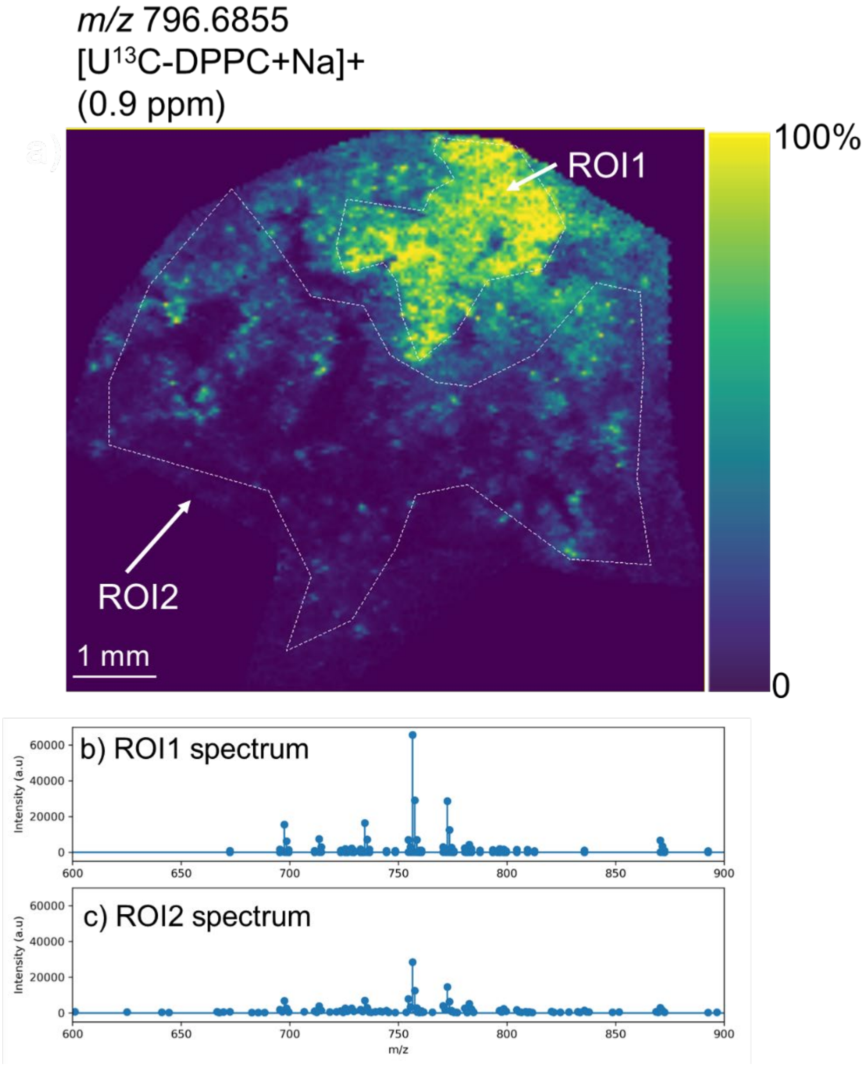

Supplementary Figure S5. (a) Ion distribution image of [U^{13}C-DPPC+Na]$^{+}$ observed at *m/z* 796.6855 in mouse lung tissue 12 h after nasal administration of CHF5633 surfactant. Signal for [U^{13}C-DPPC+Na]$^{+}$ is specific for CHF5633. (b, c) Extracted region-of-interest (ROI) spectra from the corresponding regions marked in (a) demonstrating the overall increased signal of unlabelled [PC32:0+Na]$^{+}$ in regions of CHF5633 accumulation.

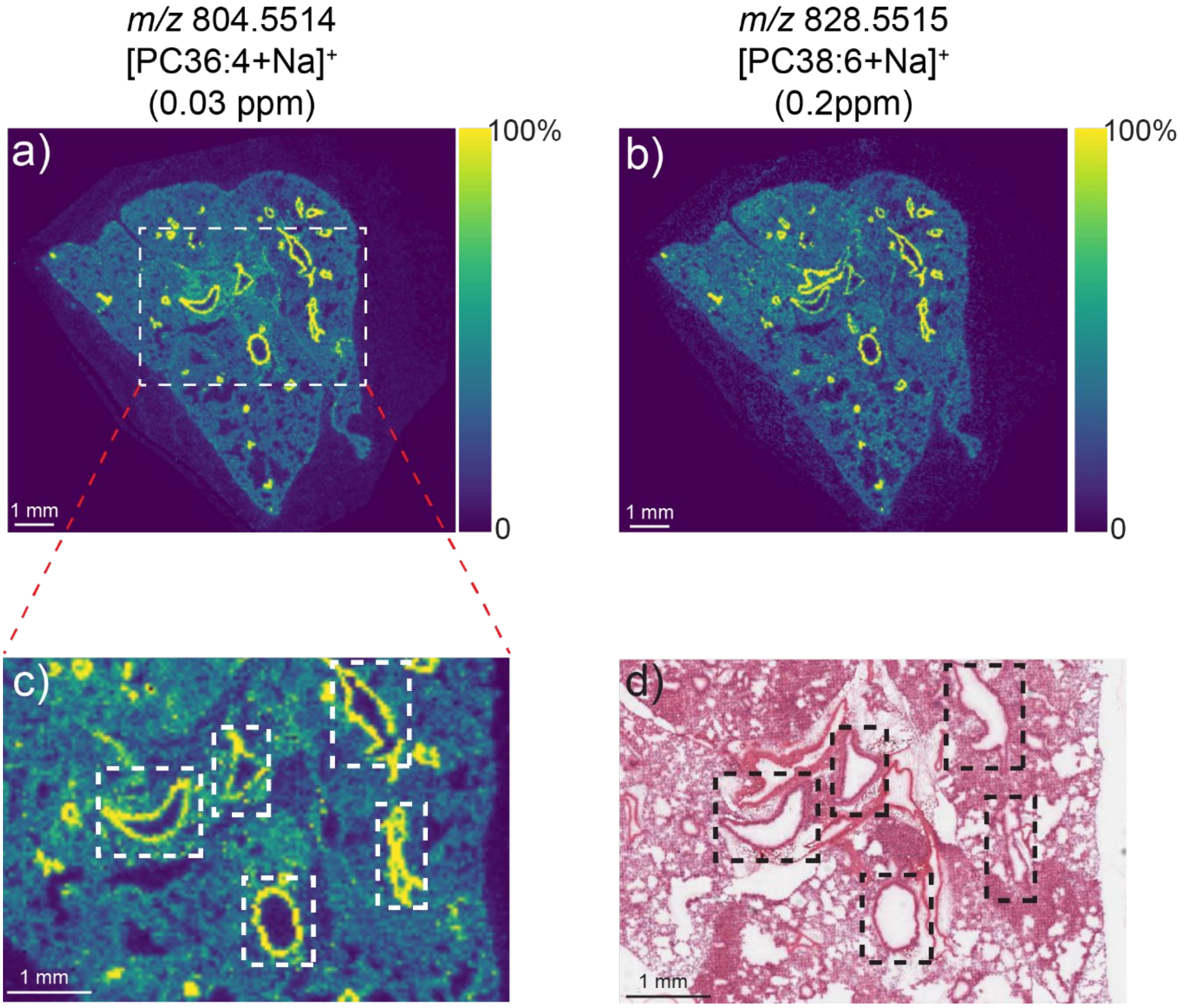

Supplementary Figure S6. Ion distribution images for (a) $[PC36:4+Na]^+$ (*m/z* 804.5514) and (b) $[PC38:6+Na]^+$ (*m/z* 828.5515) obtained from mouse lung tissue collected 6 h after administration of D_9-choline and $U^{13}C$-DPPC–containing CHF5633. Parts-per-million (ppm) mass errors are indicated in parentheses. (c) Magnification of the boxed region in (a) with selected bronchiolar regions outlined in white boxes. (d) The corresponding H&E-stained tissue section with the same selected bronchiolar regions outlined in black boxes. These data demonstrate the co-localisation of the polyunsaturated lipids PC36:4 and PC38:6 with the bronchiolar regions of the lung. All MSI images were visualised using total ion current normalisation and hotspot removal (high quantile = 99%).

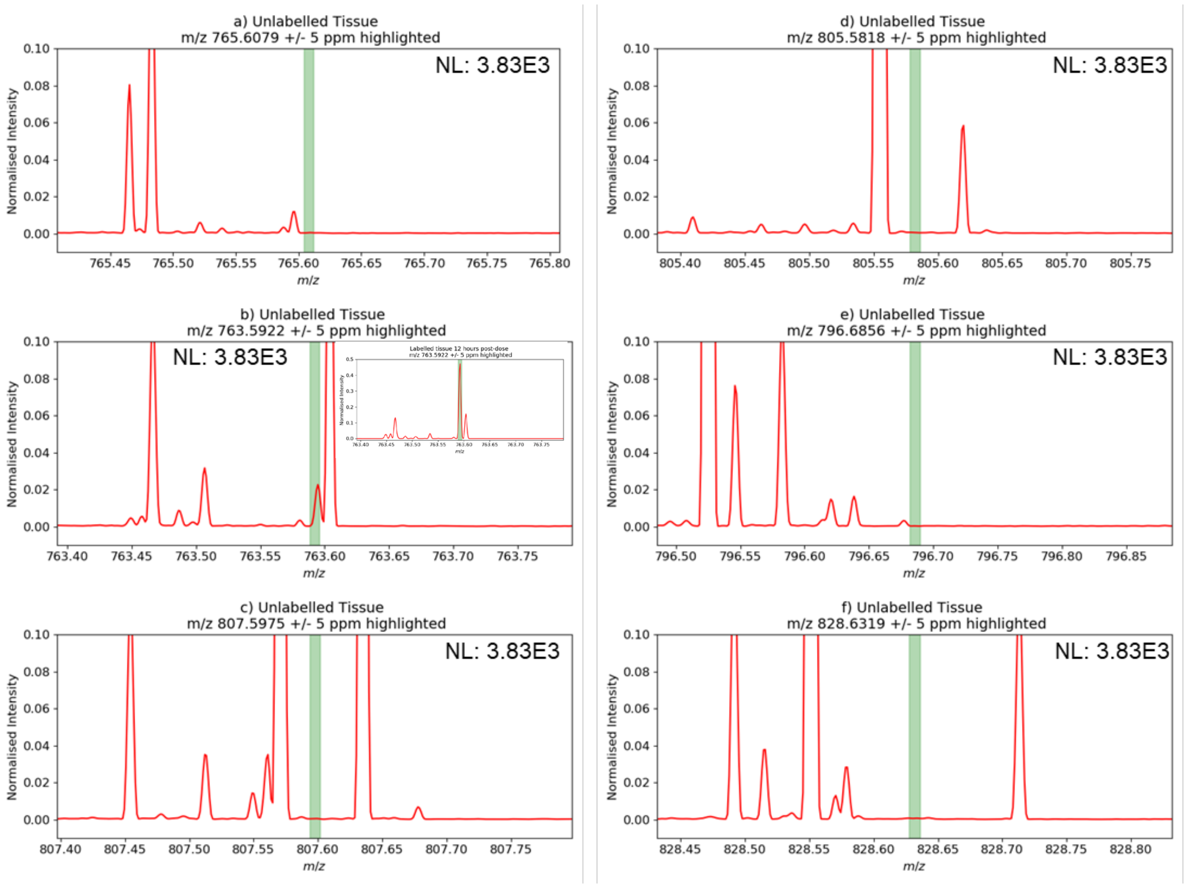

Supplementary Figure S7. Averaged MALDI mass spectra obtained from control mouse lung tissue not administered with either D$_9$-choline or U^{13}C-DPPC. The expected *m/z* ranges (±5 ppm of theoretical *m/z* value) where signal for (a) [D$_9$-PC32:0+Na]$^+$, (b) [D$_9$-PC32:1+Na]$^+$, (c) [D$_9$-PC34:1+K]$^+$, (d) [D$_9$-PC34:2+K]$^+$, (e) [U^{13}-DPPC+Na]$^+$ and (f) [^{13}C$_{24}$-PC16:0_20:4+Na]$^+$ is expected are indicated in green. Spectra are normalised to the base peak intensity (0–100 scale). Note in the case of (b) a minor signal within (±5 ppm of the expected *m/z* of [D$_9$-PC32:1+Na]$^+$ is observed. The inset in (b) shows the corresponding data collected from a mice dosed with D$_9$-choline and U-C$_{13}$-DPPC–containing CHF5633 surfactant (labels administered 12 h prior to sacrifice) showing a significantly higher signal and lower ppm mass error that supports the assignment of this signal as [D$_9$-PC32:1+Na]$^+$ in the dosed tissue (note the different y axis scales). The 'NL' value refers to the intensity of the base peak in the full range MS^1 spectrum averaged over the entire measurement region. DPPC = PC16:0/16:0.

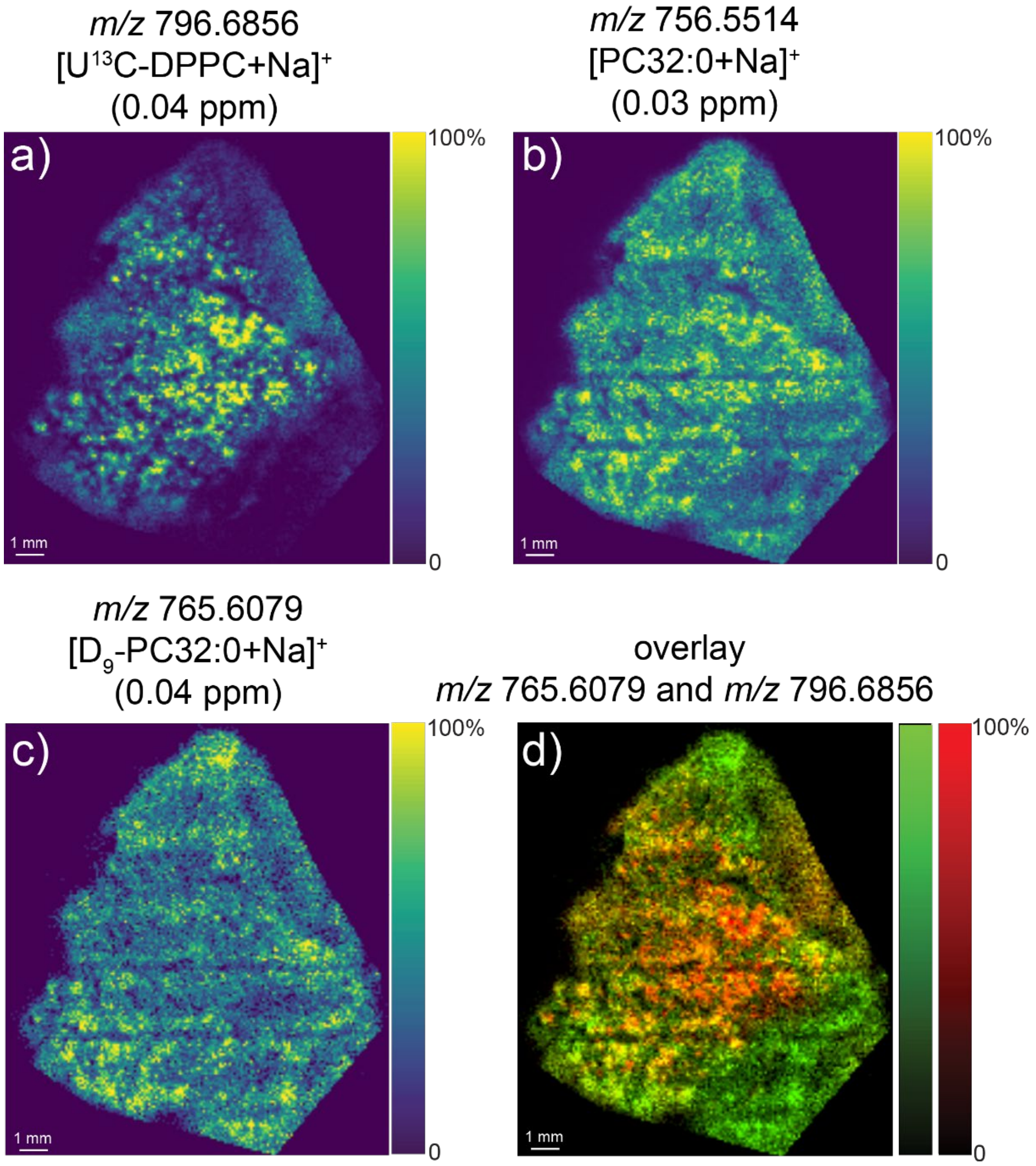

Supplementary Figure S8. MALDI-MSI data of mouse lung tissue administered with D_9-choline and U ^{13}C-DPPC–containing Poractant alfa surfactant (labels administered 18 h prior to sacrifice). Ion images of (a) *m/z* 796.6856 ([U^{13}C-DPPC+Na]$^+$), (b) *m/z* 756.5154 [PC32:0+Na]$^+$ and (c) *m/z* 765.6079 ([D_9-PC32:0+Na]$^+$). (d) Overlay image of [U-^{13}C-DPPC+Na]$^+$ (red) and [D_9-PC32:0+Na]$^+$ (green). Parts per million (ppm) mass errors are indicated in parentheses. All images were visualised using total-ion-current normalisation and using hotspot removal (high quantile = 99%). DPPC = PC16:0/16:0.

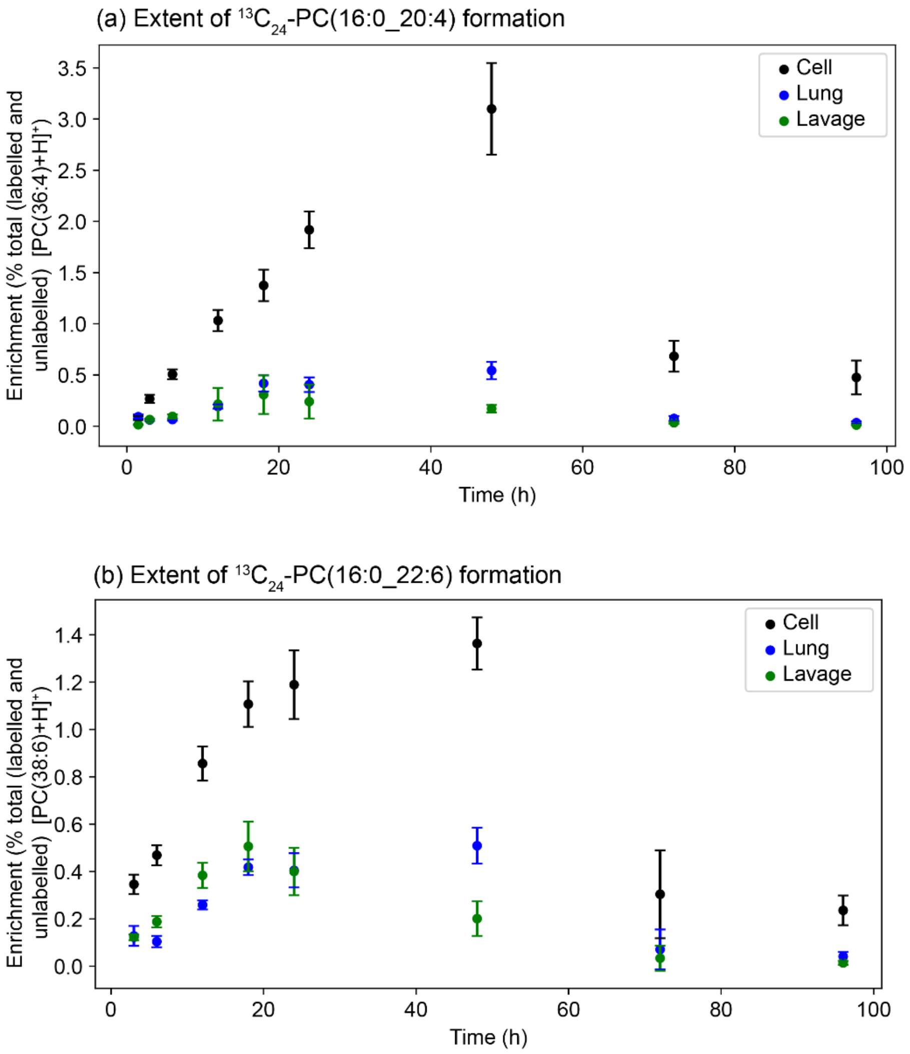

Supplementary Figure S9. ESI-MS analysis of lavage cell pellet, lung tissue and lung lavage lipid extracts collected from animals (n = 10–21 mice per time point/group) at the indicated time after nasal administration of U-^{13}C-DPPC–containing Poractant alfa surfactant. The extent of formation of acyl remodelling products (a) $^{13}C_{24}$-PC16:0_20:4 and (b) $^{13}C_{24}$-PC16:0_22:6 originating from acyl remodelling of U^{13}C-DPPC was followed over 96 hours. Error bars represent ± 1 standard deviation. $^{13}C_{24}$-labelled lipid species were detected using a precursor ion scan of m/z 189.0 for the $^{13}C_5$-labelled phosphocholine headgroup. Presented data are a subsequent analysis of data collected in reference [12]. There was a high variability in nasal delivery of surfactant to the mice in [12], with some mice receiving little surfactant. As the purpose of this figure was to present a comparison between matched sub-fractions of lung rather than comparing absolute concentrations, mice with a low abundance of delivered surfactant were excluded from this comparison, although reported in reference 12. The criterion used was an enrichment of exogenous surfactant < 0.1 % of total BALF phosphatidylcholine, based on the analysis of U^{13}C-DPPC.

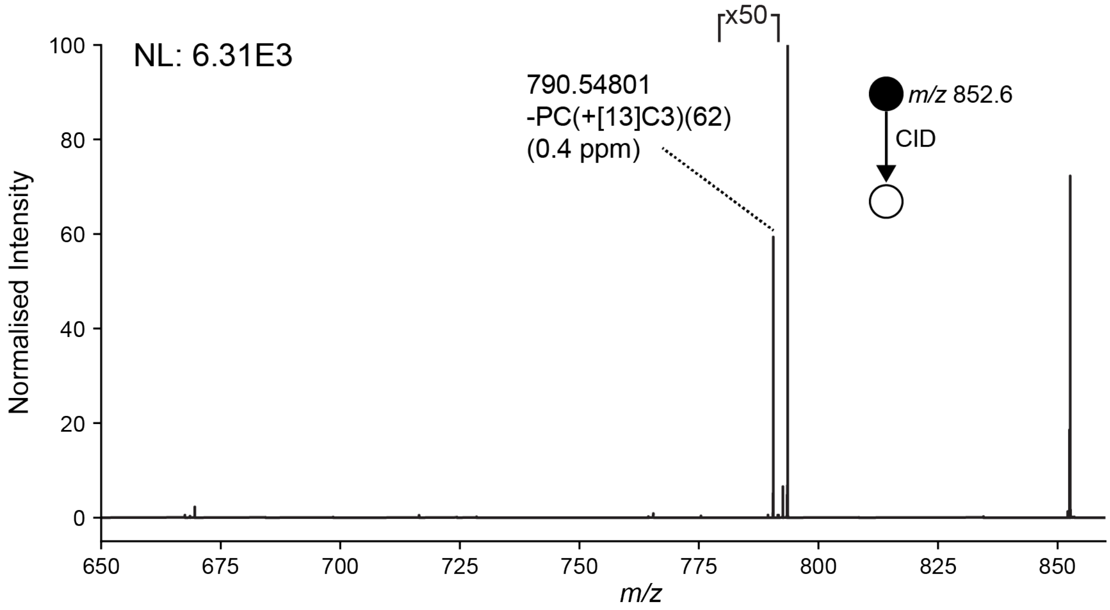

Supplementary Figure S10. MS/MS spectrum of precursor ions at *m/z* 852.6 ± 0.5 with fragment ions arising from [$^{13}C_{24}$-PC16:0_22:6+Na]$^+$ are annotated. Parts per million (ppm) mass errors are provided in parentheses. The ‘NL” value refers to the intensity of the base peak in the full range MS^1 spectrum averaged over the entire measurement region.
